# Supplementary material for: The determinants of nonprofit hospital CEO compensation
Source: PLoS One. 2024 Jul 24;19(7):e0306571. doi: 10.1371/journal.pone.0306571 (PMC11268666; doi:10.1371/journal.pone.0306571)
Supplement: S1 Appendix — (DOCX) [file pone.0306571.s001.docx]

**S1 Appendix. Supplement to the determinants of nonprofit hospital CEO compensation.**

Supporting information

**S1.1 Merging the Data**

- **Step 1)** We first match the Hospital Cost Tool (HCT) data set with the Candid facility file on hospital name. There are many misspellings and shared names in the Candid facility file, so to merge the data first we standardize the hospital names and remove any duplicate names. We then merge the subset of the HCT dataset with unique hospital names and with the subset of Candid facilities with unique hospital names and save the set of hospitals with duplicate names in each sample for the next step.
- **Step 2)** Next, we use the same process using hospital addresses instead of names and attempt the merge on the samples from the HCT and Candid that were unmatched from step 1 or had duplicate names.
- **Step 3)** Many nonprofit hospitals remain unmatched in the HCT after these two steps. We attempt to merge the remainder by hand, manually matching them with the Candid facility file. Now that each facility has a CCN attached to it, we can merge them back to the main Candid file to merge with personnel data. Some matched hospitals were types of facilities not considered in our sample, so we removed these at this point.
- **Step 4)** Due to inconsistencies in organizational structure between the form 990s and AHA dataset. A small subset of hospitals was removed from the data so we could collapse on health system. Occasionally, hospitals within a system in the AHA would be listed under an EIN used by another system in the 990s. This was likely caused by changes in hospital ownership throughout the year, given the pace of hospital mergers throughout the period. Once these inconsistencies were addressed, we collapsed by system ID and year.
- **Step 5 (Final sample)** We remove observations with no CEO compensation data, and hospitals with missing values for the financial variables we consider.

**S1.2 Identifying the CEO**

Executive compensation is reported at the EIN level. We aggregate this data to the system level where each system includes many leaders and executives with titles such as “CEO” or “President” who may be leaders of individual hospitals within the system. To identify the CEO, we use the process outlined below.

Hospitals are required to report all current officers, directors, and trustees. In addition, they also must report executive compensation information on up to 20 current employees who meet the IRS definition of a key employee (one with certain responsibilities and reportable compensation greater than $150,000 from the organization or related organizations) as well as their five highest compensated employees who are not officers, directors, trustees, or key employees.

Occasionally clinical staff members outside the scope of our research question are among the “executives” listed in the data. Because we seek to characterize executive compensation for personnel not directly involved with patient care, we limit the sample to executives that have a non-clinical role in the organization. To do so, we remove as many personnel as we can programmatically by dropping observations with titles reflecting direct patient care. Occasionally clinical personnel will be the highest paid employee listed on a 990 form. There were 15,969 clinical personnel dropped due to titles indicating a clinical position. Appendix exhibit A.3 presents all the titles excluded, including common misspellings.

In addition to clinical staff, there are university presidents, head football coaches, and other academic positions not associated with the hospital that are reported on form 990s for university hospitals. For university hospitals, we manually checked and removed academic personnel. This step dropped 188 observations in the personnel file.

The IRS requires compensation from “related organizations” to be reported on 990 forms. This requirement often leads to system level executives being reported on 990 forms of multiple hospitals within the system. So, to drop the duplicates within system, we drop all observations that are duplicates in system, year, name, and total compensation. There are many misspellings in the 990 forms, so we manually inspect the set of observations that are duplicates in total compensation, system, and year and remove duplicates not caught in the first step due to a misspelling in name. Next, there are the set of duplicates that are duplicates in name, system, and year but not total compensation (executives listed under two E-INs but with different compensation levels). To handle these observations, we make the following rule. If the duplicates are reported under the same EIN, we add the compensation values together (this is rare). If they are reported under different EINs we include the larger compensation value.

Finally, now that we have removed the duplicate personnel and executives outside the scope of our paper, we use the titles reported in the 990 form to flag leaders that are likely to be the CEO. The flag is then adjusted at the system level to account for errors we were able to identify manually inspecting the data. After the possible leaders have been flagged, we designate the system level “CEO” as the highest paid executive who is flagged as a possible leader.

**S1.3 Exclusion Criteria for removing clinicians from the sample**

| **Includes** | **Does not Include** | **Observations dropped** |
| --- | --- | --- |
| NEU | CEO, COO, CFO, EXEC, TREASURER, SECRE, EXEC | 132 |
| CARD | CEO, COO, CFO, TREASURER, SECRE, EXEC | 370 |
| RADI | CEO, COO, CFO, TREASURER, SECRE, EXEC | 201 |
| ANES | CEO, COO, CFO, TREASURER, SECRE, EXEC | 360 |
| INTENS | CEO, COO, CFO, TREASURER, SECRE, EXEC | 17 |
| SURG | CEO, COO, CFO, TREASURER, SECRE, EXEC | 845 |
| PLUM | CEO, COO, CFO, TREASURER, SECRE, EXEC | 11 |
| PEDIA | CEO, COO, CFO, TREASURER, SECRE, EXEC | 48 |
| PHYS, (or PHSY) | CEO, COO, CFO, TREASURER, SECRE, EXEC | 9,136 |
| GASTRO | CEO, COO, CFO, TREASURER, SECRE, EXEC, PRESIDENT | 24 |
| IMMUN | CEO, COO, CFO, TREASURER, SECRE, EXEC, PRESIDENT | 2 |
| DERM | CEO, COO, CFO, TREASURER, SECRE, EXEC, PRESIDENT | 11 |
| BASKET | CEO, COO, CFO, TREASURER, SECRE, EXEC, PRESIDENT | 5 |
| BOARD MEMBER | CEO, COO, CFO, TREASURER, SECRE, EXEC, PRESIDENT | 1,883 |
| UROLO | CEO, COO, CFO, TREASURER, SECRE, EXEC, PRESIDENT | 34 |
| FOOTBALL | CEO, COO, CFO, TREASURER, SECRE, EXEC, PRESIDENT | 7 |
| OGIST | CEO, COO, CFO, TREASURER, SECRE, EXEC, PRESIDENT | 136 |
| PSYCH | CEO, COO, CFO, TREASURER, SECRE, EXEC, PRESIDENT | 106 |
| DOCTOR | CEO, COO, CFO, TREASURER, SECRE, EXEC, PRESIDENT | 176 |
| HOSPTALIST | CHIEF, DIR, VP, APNP | 16 |
| FAMILY | CEO, CFO, COO, TREASURER, SEC, EXEC, PRESIDENT, DIR, CHAIR, CHF, CHIEF, NURSE, VP CHAIRMAN | 43 |
| INTERNIST | CEO, COO, CFO, TREASURER, SECRE, EXEC, PRESIDENT | 7 |
| “ MD” | CEO, COO, CFO, TREASURER, SECRE, EXEC, PRESIDENT | 5 |
| **The following have no exclusion criteria, if the following title is listed, the personnel are dropped** | | |
| MEDICAL DOCT, PHARMACIST,HARMACIST, ORTHO SPECIALIST, EMERGENCY ROOM MD, MD, PHYSCIAN, ORTHOPEDIST, MD ENT, STAFF MD ORTHO, PHUYSICIAN, DO, DENTIST, ATTENDING PH, EMERGENCY/URGENT CARE, ER PA, ONCOLOGY/HEMOTOLOGY, OB/GYN, PA-C, PHYISICNA, ORTHOPEDIC S, ORTHOPEDIC TRAUMA, SPINE & SCOLIOSIS, PHYISICIAN, ORTHOPEDIC M, PHARMACIST TEAM LEAD, PHYSICIAN, ER PHYXICIAN, PAIN MANAGEMENT PHYXICIAN, OBGYN, OBSTETRICS/GYNECOLOGIST, MEDICAL PROVIDER, OBSTERTICS & GYNECOLOGY, EMPLOYED M D, MD/ONCOLOGY, NEONATOLOGISTS, PODIATRIST, ER PROVIDER, GERIARTIC, OBSTETRICS, ORTHOPEDIC, ORTHOPEDIC PA, ORTHOPEDIC SPECIALIST, ORTHO SPECIALISTS, ENT SPECIALIST, OBSTETRICIAN, HOSPITALIST, HOSPITALIST MD, ADULT HOSPITALIST, OB GYNE HOSPITALIST, HOSPITALIST INTERNAL MEDICINE, PAYSICIAN, GEN PRAC PHY, PSHCYIATRIST, PHYCHIATRIST-CMHC, ER MEDICINE, PT MANAGER, MD/ONCOLOGY, M.D.,D O, ED HYSICIAN, EMERGENCY MEDICINE, THERAPIST, MEDICAL DR, ORTHO, PL1YSICUN, PERFUSIONIST, LABATORY MANAGER, EMERGENCY SERVICES, PA, HOSPTALIST, MD-INTERNAL MEDICI, INTERMIST, MD, GENERAL MEDICINE, INTERNAL MED, INTERNAL MED-GENERAL, INTERNAL MEDICINE, PYSICIAN ADVANCED CLINICIAN. | | 1,290 |
| **The following criteria is how we identified nurses from the sample. To be dropped they must also have had a salary below $250,000.** | | |
| APRN | TREASURER, SECRE, DIR | 27 |
| A R N P, LPN, NP, ADVANCED PRACTICE RN – CERT NP, CNP |  | 35 |
| NURSE PRACT | CEO, COO, CFO, EXEC, TREASURER, SECRE, VP, VICE, V.P., V P, DIRECTOR, CNO, ADM | 192 |
| REGISTERED | CEO, COO, CFO, EXEC, TREASURER, SECRE, VP, VICE, V.P., V P, DIRECTOR, CNO, ADM, DIRECTOR | 170 |
| RN | SECRE, TREAS, CHIEF, DON, DIR, PRES, VP, ATTORNEY, GOVERN, CHAIR, COO-, AUD, ADMIN, CEO, CE/, CFO, CMO, INTERNAL MED. | 575 |
| RN, R.N., R N, ANP, APN |  | 32 |
| FNP | COO | 14 |
| CRNA | CEO, COO, CFO, EXEC, TREASURER, SECRE | 209 |
| HOME HEALTH PER VISIT PT, HOME HEALTH PT, PT |  | 5 |
| NURSE | CEO, COO, CFO, EXEC, TREASURER, SECRE, VP, VICE, V.P., V P, CHIEF, CNO, ADMIN, DIR, MANAGER, MGR | 109 |
| MURSED, ADVANCED |  | 10 |

**S1.4**

|  | Untransformed Profits ($ Millions) | | | | Cube Root Transformation on Profit | | | |
| --- | --- | --- | --- | --- | --- | --- | --- | --- |
|  | (1) | | (2) | | (3) | | (4) | |
|  | 2012 | 2019 | 2012 | 2019 | 2012 | 2019 | 2012 | 2019 |
| Profit ($ mill) | 0.0015*** | 0.0009*** | 0.0009*** | 0.0006*** | 0.0039*** | 0.0020*** | 0.0034*** | 0.0018*** |
| Charity Care ($mill) | . | . | 0.0021* | 0.0009 | . | . | 0.0018** | 0.0012 |
| >0 and <50% Teaching | . | . | 0.3344* | 0.4621*** | . | . | 0.1684 | 0.3415** |
| >50% Teaching | . | . | 0.7212*** | 0.7028*** | . | . | 0.3679*** | 0.5208*** |
| **Number of Hospitals** |  |  |  |  |  |  |  |  |
| Two to Three | 0.8020*** | 0.8609*** | 0.6879*** | 0.7609*** | 0.4160*** | 0.4695*** | 0.3738*** | 0.4226*** |
| Four to Nine | 0.7921*** | 1.1674*** | 0.7375*** | 1.0263*** | 0.2919*** | 0.6891*** | 0.2487** | 0.5942*** |
| More than 10 | 0.4053* | 0.9530*** | 0.4733* | 0.8961*** | -0.0056 | 0.6314*** | -0.1481 | 0.4482** |
|  |  |  |  |  |  |  |  |  |
|  | **Untransformed Profits ($ Millions)** | | | | **Cube Root Transformation on Profit** | | | |
|  | (1) | | (2) | | (3) | | (4) | |
|  | 2012 | 2019 | 2012 | 2019 | 2012 | 2019 | 2012 | 2019 |
| Profit ($ mill) | 0.0008*** | 0.0005*** | 0.0006*** | 0.0005*** | 0.0023*** | 0.0011*** | 0.0021*** | 0.0010*** |
| Charity Care ($mill) | . | . | 0.0008 | -0.0001 | . | . | 0.0009 | 0.0008 |
| >0 and <50% Teaching | . | . | 0.2793* | 0.3092** | . | . | 0.2001 | 0.2909** |
| >50% Teaching | . | . | 0.3300*** | 0.2090* | . | . | 0.2667*** | 0.2107* |
| **Number of Beds** |  |  |  |  |  |  |  |  |
| 100 to 299 | 0.9009*** | 0.9894*** | 0.8920*** | 0.9894*** | 0.7504*** | 0.8699*** | 0.7525*** | 0.8794*** |
| 300 to 499 | 1.271*** | 1.2583*** | 1.2133** | 1.2362*** | 0.9634*** | 1.0475*** | 0.9311*** | 1.0378*** |
| More than 500 | 1.568*** | 1.8062*** | 1.4419*** | 1.7049*** | 1.1426*** | 1.5405*** | 1.0291*** | 1.4137*** |
|  |  |  |  |  |  |  |  |  |
|  | **Untransformed Profits ($ Millions)** | | | | **Cube Root Transformation on Profit** | | | |
|  | (1) | | (2) | | (3) | | (4) | |
|  | 2012 | 2019 | 2012 | 2019 | 2012 | 2019 | 2012 | 2019 |
| Profit ($ mill) | 0.0007*** | 0.0005*** | 0.0004** | 0.0004*** | 0.0018*** | 0.0007*** | 0.0015*** | 0.0006*** |
| Charity Care ($mill) | . | . | 0.0010 | 0.0000 | . | . | 0.0012* | 0.0010*** |
| >0 and <50% Teaching | . | . | 0.3057* | 0.3608** | . | . | 0.2539* | 0.3723*** |
| >50% Teaching | . | . | 0.4099*** | 0.3144*** | . | . | 0.3391*** | 0.3160*** |
| **Number of Discharges** |  |  |  |  |  |  |  |  |
| 5,000 to 19,999 | 0.7808*** | 0.7531*** | 0.7773*** | 0.7543*** | 0.6558*** | 0.6779*** | 0.6708*** | 0.6923*** |
| 20,000 to 99,999 | 1.4880*** | 1.5525*** | 1.4207*** | 1.5113*** | 1.2013*** | 1.3968*** | 1.1748*** | 1.3742*** |
| More than 100,000 | 1.9745*** | 2.1474*** | 1.8160*** | 1.9865*** | 1.5876*** | 2.0295*** | 1.4223*** | 1.8077*** |

Appendix Exhibit S1.4 presents the coefficient estimates from the regression analysis. The coefficients presented represent the estimated association between log CEO compensation (in 2019 dollars) and profit and charity care (in $millions), teaching status, and our categorized measures of hospital bed size, number of hospitals, and adjusted discharges. Teaching status is defined as the percentage of adjusted discharges that occurred at a teaching hospital within the system or hospital. The coefficients of profit and charity care can be interpreted as the estimated percentage change in CEO compensation associated with a one million dollar increase in profit or charity care. The coefficients on the size categorical variables should be interpreted as the increase in CEO compensation relative to the reference group associated with a hospital in each size category. In specifications three and four, we perform a cube root transformation on profit. To be able to interpret the coefficients on profit as the estimated percentage change in CEO compensation from a one million dollar increase in profit, we back-transform the coefficients on the profit covariate where we used the cube-root transformation. The stars ***,**,* represent 99.9,99, and 95 percent confidence levels.

**S1.5 Oaxaca Decomposition**

|  | | Mean | | Coefficient | | | | Mean Explained Effect^1^ | Coefficient Unexplained Effect^2^ | |
| --- | --- | --- | --- | --- | --- | --- | --- | --- | --- | --- |
|  | 2012 | | 2019 | | 2012 | 2019 |  | | |  |
| Profit ($Mill) | 94.65 | | 149.83 | | 0.0006 | 0.0005 | 0.0257 | | | -0.0088 |
| Charity Care ($ Mill) | 15.14 | | 17.18 | | 0.0008 | -0.0001 | -0.0001 | | | -0.0126 |
| >0 and <50% Teaching | 0.03 | | 0.06 | | 0.2793 | 0.3092 | 0.0092 | | | 0.0009 |
| >50% Teaching | 0.09 | | 0.8 | | 0.3300 | 0.2090 | -0.0025 | | | -0.0106 |
| 100 to 299 Beds | 0.28 | | 0.23 | | 0.8919 | 0.9894 | -0.0477 | | | 0.0274 |
| 300 to 499 Beds | 0.114 | | 0.106 | | 1.2133 | 1.2362 | -0.0010 | | | 0.0026 |
| 500 Beds or more | 0.18 | | 0.22 | | 1.4418 | 1.7049 | 0.0692 | | | 0.0475 |

1. Mean “explained” effect can be expressed as (Mean 2019- Mean 2012)*(Coefficient in 2019)
2. Coefficient “Unexplained” effect can be expressed as (Coefficient 2019-Coefficient 2012)*Mean 2012

The Oaxaca decomposition has long been used by labor economists to break down differences in mean wages between two groups (e.g. males vs. females) into the portion that is attributable to differences in pay for people with the same characteristics in each group, versus differences in pay resulting from differences in the characteristics of people in these two groups. We can apply the decomposition to break down differences in CEO compensation that occurred due to changes in the means of covariates in our analysis and changes in the coefficients.

Begin by estimating regressions of allowed amounts on patient characteristics for the years 2012 and 2019.

$${CEO}_{12}= \alpha_{0}+ \alpha_{1}{Prof}_{12}+ \alpha_{2}{CC}_{12}+\alpha_{3} {Teaching}_{12}+\alpha_{4}{Bed}_{12}+\varepsilon_{12}$$

$${CEO}_{19} = \beta_{0}+ \beta_{1}{Prof}_{19}+ \beta_{2}{CC}_{19}+\beta_{3}{Teaching}_{19}+\beta_{4}{Bed}_{19}+\varepsilon_{19}$$

Subscripts on variables refer to the year 2012 or 2019.

*CEO*= Log of CEO compensation in 2019 dollars.

*α* is used to represent coefficients in 2012 equation

*β* is used to represent coefficients in 2019 equation

*Prof* =Profit in millions of 2019 dollars

*CC*= Charity care cost in millions of 2019 dollars.

*Teaching* is a vector of categorical variables for teaching status

*Bed* is a vector of categorical variables for levels of bed size

The overall change in the mean log CEO compensation between 2012 and 2019 can be expressed as the following where ***X*** is a vector of all covariates and *α_12_* and *β_19_* are vectors of the coefficients in each year.

$$\bar{CEO}_{19}- \bar{CEO}_{12}=({\beta_{0}-a}_{0})+\beta_{19}\bar{X}_{19}+{\beta_{19}\bar{X}}_{12}- \beta_{19}\bar{X}_{12}- a_{12}\bar{X}_{12}$$

Because the average value of residuals in a linear regression is zero, the difference in log CEO compensation between 2019 and 2012 includes only coefficients and means of explanatory variables. These coefficients and mean explanatory variables can be arranged to yield the following, which breaks down the change in log CEO compensation from 2019 to 2012 into three components, the amount of the change explained by (1) the difference in the intercepts, (2) changes in the coefficients, and (3) changes in the means of the covariates:

$$\bar{CEO}_{19}- \bar{CEO}_{12}= \underset{Changes in Intercept}{\underbrace{\left( \beta_{0}-a_{0} \right)}}+\underset{\begin{aligned} \\ Changes in Coefficients \end{aligned}}{\underbrace{\bar{X}_{12} \left( \beta_{19}-a_{12} \right)}}+\underset{Change in Covariates}{\underbrace{(\bar{X}_{19}- \bar{X}_{12})\beta_{19}}}$$

These three components are mutually exclusive and exhaustive, comprising the entire difference of mean log CEO compensation.

**S1.6 Cross-Tabulation For Number of Beds and Number of Hospitals**

|  | Single Hospital | Two or Three Hospitals | Four to Nine Hospitals | Ten or more hospitals | Total |
| --- | --- | --- | --- | --- | --- |
| Under 100 Beds | 842 | 9 | 1 | 0 | 852 |
| 100-299 Beds | 440 | 66 | 11 | 0 | 517 |
| 300-499 Beds | 127 | 73 | 17 | 2 | 219 |
| 500 or more Beds | 48 | 109 | 160 | 76 | 393 |
| Total | 1457 | 257 | 189 | 78 |  |

Appendix Exhibit S1.6 presents a cross tabulation for the categorical variables of number of hospitals and number of beds.

**S1.7 Robustness Check for Joint Significance**

|  | | **Coefficient** | **P - Value** |
| --- | --- | --- | --- |
| 2019 Year Dummy | | 0.0723 | 0.095 |
| Profit ($Millions) | | 0.0006 | 0.000 |
| 2019#Profit | | -0.0001 | 0.590 |
| Charity Care ($Millions) | | 0.0008 | 0.246 |
| 2019#Charity Care | | -0.0008 | 0.379 |
| >0% and <50% Teaching | | 0.2793 | 0.032 |
| 2019#>0% and <50% Teaching | | 0.0299 | 0.862 |
| >50 % Teaching | | 0.3300 | 0.000 |
| 2019# >50% Teaching | | -0.1210 | 0.315 |
| 100 to 299 Beds | | 0.8920 | 0.000 |
| 2019# 100 to 299 Beds | | 0.0974 | 0.172 |
| 300 to 499 Beds | | 1.2133 | 0.000 |
| 2019#300 to 499 Beds | | 0.0229 | 0.816 |
| Over 500 Beds | | 1.4419 | 0.000 |
| 2019#Over 500 Beds | | 0.2630 | 0.012 |
| Constant | | 12.5649 | 0.000 |
| **N** | **1,981** | | |
| **Wald Test*** | **F( 8, 1965) = 3.23, P-Value = .0012** | | |

Appendix Exhibit S1.7 presents the coefficient estimates from the regression analysis including interaction terms with the independent variables from the main specifications and their interaction with a 2019-year dummy. CEOs from 2019 and 2012 are included in this analysis. The coefficients presented represent the estimated association between log CEO compensation (in 2019 dollars) and profit and charity care (in $millions), teaching status, and our categorized measures of hospital bed size, a 2019-year dummy, and all variables interacted with the 2019-year dummy. Teaching status is defined as the percentage of adjusted discharges that occurred at a teaching hospital within the system or hospital. The coefficient on the 2019 base year can be interpreted as the relative increase to the base case CEO in 2019 relative to 2012. The base case CEO is a CEO of a hospital or health system with no adjusted discharges at a teaching hospital, less than 100 beds, and zero profit and charity care.

*Wald test statistic is presented for the subset of variables including 2019 year dummy, and all variables interacted with 2019 year dummy
